# Caspofungin Treatment of Aspergillus fumigatus Results in ChsG-Dependent Upregulation of Chitin Synthesis and the Formation of Chitin-Rich Microcolonies

**DOI:** 10.1128/AAC.00862-15

**Published:** 2015-09-18

**Authors:** Louise A. Walker, Keunsook K. Lee, Carol A. Munro, Neil A. R. Gow

**Affiliations:** Aberdeen Fungal Group, School of Medical Sciences, Institute of Medical Sciences, University of Aberdeen, Foresterhill, Aberdeen, United Kingdom

## Abstract

Treatment of Aspergillus fumigatus with echinocandins such as caspofungin inhibits the synthesis of cell wall β-1,3-glucan, which triggers a compensatory stimulation of chitin synthesis. Activation of chitin synthesis can occur in response to sub-MICs of caspofungin and to CaCl_2_ and calcofluor white (CFW), agonists of the protein kinase C (PKC), and Ca^2+^-calcineurin signaling pathways. A. fumigatus mutants with the *chs* gene (encoding chitin synthase) deleted (Δ*Afchs*) were tested for their response to these agonists to determine the chitin synthase enzymes that were required for the compensatory upregulation of chitin synthesis. Only the Δ*AfchsG* mutant was hypersensitive to caspofungin, and all other Δ*Afchs* mutants tested remained capable of increasing their chitin content in response to treatment with CaCl_2_ and CFW and caspofungin. The resulting increase in cell wall chitin content correlated with reduced susceptibility to caspofungin in the wild type and all Δ*Afchs* mutants tested, with the exception of the Δ*AfchsG* mutant, which remained sensitive to caspofungin. *In vitro* exposure to the chitin synthase inhibitor, nikkomycin Z, along with caspofungin demonstrated synergistic efficacy that was again *Af*ChsG dependent. Dynamic imaging using microfluidic perfusion chambers demonstrated that treatment with sub-MIC caspofungin resulted initially in hyphal tip lysis. However, thickened hyphae emerged that formed aberrant microcolonies in the continued presence of caspofungin. In addition, intrahyphal hyphae were formed in response to echinocandin treatment. These *in vitro* data demonstrate that A. fumigatus has the potential to survive echinocandin treatment *in vivo* by *Af*ChsG-dependent upregulation of chitin synthesis. Chitin-rich cells may, therefore, persist in human tissues and act as the focus for breakthrough infections.

## INTRODUCTION

Cases of invasive aspergillosis are associated with high mortality rates of around 70% to 90% in immunocompromised patients (1). The majority of these infections are caused by Aspergillus fumigatus and Aspergillus lentulus (2–6), with the remaining due to Aspergillus flavus (10%), Aspergillus niger (2%), and Aspergillus terreus (2%) (6–8). There are few antifungal agents that are effective for treatment of invasive aspergillosis, and some classes of antifungals that are effective against other fungal pathogens are not able to control Aspergillus infections. In addition, recent case histories have shown examples of emerging antifungal drug resistance in Aspergillus clinical isolates (6, 9, 10) and associated skin carcinomas in some patients treated with voriconazole (11). The echinocandins have been shown to cause lysis of growing hyphal tips but are considered to be fungistatic against molds (12). Because treatment with the echinocandins fails to completely inhibit growth of Aspergillus species, it is difficult to determine clear endpoints for inhibition and accurate MICs (13). As a result, alternative methods, such as the minimum effective concentration (MEC), have been introduced to determine the activity of echinocandins against filamentous fungi. The MEC is defined as the lowest drug concentration at which short, stubby, highly branched hyphae are observed (13–17). Treatment of A. fumigatus with the echinocandin, caspofungin, leads to lysis of hyphal tips that is a result of inhibition of apical A. fumigatus Fks1 (*Af*Fks1)—the targeted β-1,3-glucan synthase (12, 18). Although treatment with caspofungin results in lysis of hyphal tips, viability staining has shown that older subapical compartments of A. fumigatus hyphae can remain viable after exposure to caspofungin (12).

Previously, treatment of Candida albicans with caspofungin has been shown to lead to a compensatory increase in cell wall chitin synthesis that results in restoration of cell wall integrity (19). Candida tropicalis, Candida parapsilosis, and Candida guilliermondii but not Candida glabrata also demonstrated compensatory upregulation of chitin content in response to treatment with caspofungin (20–22). In C. albicans, the protein kinase C (PKC), high-osmolarity glycerol (HOG), and Ca^2+^-calcineurin signaling pathways all contribute to the regulation of transcriptional activation of chitin synthesis (23). In this organism, there are only four Chs enzymes: Chs2 and Chs8 (class I), Chs1 (class II), and Chs3 (class IV). Treatment of C. albicans with a combination of CaCl_2_ and calcofluor white (CFW) stimulated the Ca^2+^-calcineurin and PKC signaling pathways, respectively, and led to a 3- to 4-fold increase in chitin content. Strains of C. albicans that have elevated chitin contents were less susceptible to caspofungin than cells with wild-type chitin levels (19, 22, 24, 25).

The A. fumigatus cell wall is comprised of 20% chitin, which is synthesized by eight Chs enzymes: A. fumigatus ChsA (*Af*ChsA), *Af*ChsB, *Af*ChsC, *Af*ChsD, *Af*CsmA (*Af*ChsE), *Af*ChsF, *Af*ChsG, and *Af*CsmB (2, 3, 26–29), which by sequence homology fall into different classes and have been characterized extensively via the analysis of single and multiple mutants. The class III and V to VII chitin synthase enzymes are specific to filamentous fungi. Disruption of single chitin synthase genes to create Δ*AfchsA* (class I), Δ*AfchsB* (class II), and Δ*AfchsC* (class III) mutants resulted in mild or no phenotypic growth effects compared to the wild type (26, 29–31). In contrast, hyphae of the Δ*AfchsD* (class VI) mutant were shown to have an increase in chitin content, and the Δ*AfchsF* mutant had a 25% reduction in chitin compared to the wild type (29). Disruption of the class V enzyme, *AfcsmA*, resulted in an 80% reduction in conidial chitin content (29), and disruption of *AfcsmA* and *AfcsmB* (class VII) resulted in hypersensitivity to caspofungin (28). The Δ*AfcsmA* and Δ*AfcsmB* mutants also had a defect in conidiation that may be abrogated by growth in osmotically stabilized media (28, 29, 32). A quadruple Δ*AfcsmA* Δ*AfcsmB* Δ*AfchsF* Δ*AfchsD* mutant was significantly attenuated in immunosuppressed mice (29). The Δ*AfchsG* single mutant and a quadruple Δ*AfchsA* Δ*AfchsC* Δ*AfchsB* Δ*AfchsG* mutant were hyperbranched and had reduced radial growth (26, 29). The Δ*AfchsA* Δ*AfchsC* Δ*AfchsB* Δ*AfchsG* mutant was also shown to have a reduction in conidiation, and conidia that were produced had a disorganized melanin layer on the surface which was attached loosely to the inner cell wall. *Af*ChsG was also shown to be required for *in vitro CHS* enzyme activity and was involved in synthesizing chitin in the conidial wall (29). However, the quadruple Δ*AfchsA* Δ*AfchsC* Δ*AfchsB* Δ*AfchsG* mutant was as virulent as the wild type in a murine model of pulmonary aspergillosis (29). The double class III/class V Δ*AfchsG* Δ*AfchsE* mutant had a 50% reduction in chitin content compared to wild-type cells and a 95% reduction in chitin synthase enzyme activity (31).

The aims of this work were to determine whether treatment of A. fumigatus with agents that increased chitin content affected susceptibility to caspofungin and to establish which Chs enzymes were important for the chitin upregulation in response to caspofungin. The data demonstrate that hyphae with high chitin could survive caspofungin treatment and that this response was strongly *AfCHSG* dependent.

## MATERIALS AND METHODS

### Strains, media, and growth conditions.

A. fumigatus strains used in this study are listed in Table 1. A. fumigatus strains were maintained on Sabouraud dextrose (Sabdex) agar plates (1% mycological peptone [wt/vol], 4% glucose [wt/vol], and 2% agar [wt/vol]).

**TABLE 1:** A. fumigatus strains used in this study

Strain	Parental strain	Genotype	Source or reference no.
H-237		Wild type	Clinical isolate
H-458	H-237	Δ*chsC*	26
H-452	H-237	Δ*chsD*	27
H-480	H-237	Δ*chsG*	26
H-466	H-458	Δ*chsC* Δ*chsB*	Unpublished
H-484	H-458	Δ*chsC* Δ*chsG*	31

### Antifungal agents.

Cells were grown in RPMI 1640 supplemented with the following inhibitors: 2 μg/ml caspofungin (obtained from Aberdeen Royal Infirmary Pharmacy) and 2 μg/ml nikkomycin Z (Sigma-Aldrich, United Kingdom), which were dissolved in sterile water. In some experiments, A. fumigatus was pretreated by growing in Sabdex broth containing 0.2 M CaCl_2_ and 100 μg/ml CFW (Sigma-Aldrich, United Kingdom) for 8 h at 37°C with shaking at 200 rpm to elevate the chitin content of hyphal cells.

### Caspofungin sensitivity testing on solid medium.

Caspofungin was incorporated into RPMI 1640 agar plates at 2 μg/ml and 4 μg/ml. In some experiments, caspofungin was used in combination with 2 μg/ml nikkomycin Z. A. fumigatus spores were collected and serially diluted to generate suspensions containing 1 × 10^6^ to 1,000 spores/ml in sterile water. Plates were inoculated with 3-μl drops of each spore suspension and incubated for 48 h at 37°C.

### Antifungal susceptibility testing.

MICs were determined by broth microdilution testing using the CLSI (formerly NCCLS) guideline M38-A2 for filamentous fungi (33). Drug concentrations ranged from 0.032 μg/ml to 16 μg/ml of caspofungin. Caspofungin was serially diluted with sterile water in flat-bottomed 96-well plates. A. fumigatus spores were collected from agar plates in phosphate-buffered saline (PBS) (Oxoid) plus 0.1% Tween 80 (Sigma) and inoculated in 11 ml 2× RPMI 1640, and 200 μl of culture was added to each well. Plates were incubated for 48 h at 37°C. After incubation, each well was mixed thoroughly and optical densities were read in a VersaMax tunable microplate reader (Molecular Devices, CA, USA) at 405 nm.

### Determination of dry weights of mycelia.

Dry weights of wild-type and Δ*Afchs* mutants were determined after 24 h growth in RPMI 1640 broth alone or supplemented with 2 μg/ml caspofungin. After incubation, cultures were collected and filtered through preweighed 0.45-μm filters. The filters containing A. fumigatus strains were dried at 80°C for 24 h and were then pared to constant weight.

### Fluorescence microscopy.

After washing with sterile water to remove any excess medium, samples were fixed in 10% (vol/vol) neutral buffered formalin (Sigma-Aldrich, United Kingdom) and examined by phase differential interference contrast (DIC) microscopy. Cells were stained with 25 μg/ml CFW to visualize chitin. All samples were examined by fluorescence microscopy using a Zeiss AxioPlan 2 microscope. Images were recorded digitally using the OpenLAB system (OpenLAB v4.04; Improvision, Coventry, United Kingdom) using a Hamamatsu C4742-95 digital camera (Hamamatsu Photonics, Hamamatsu, Japan).

### Electron microscopy.

Cultures were harvested by centrifugation, and the pellets were fixed in 2.5% (vol/vol) glutaraldehyde in 0.1 M sodium phosphate buffer (pH 7.3) for 24 h at 4°C. Samples were encapsulated in 3% (wt/vol) low melting point agarose prior to embedding in Spurr's resin following a 24-h processing schedule on a Lynx tissue processor (secondary 1% OsO_4_ fixation, 1% uranyl acetate contrasting, ethanol dehydration, and infiltration with acetone/Spurr resin). Additional infiltration was provided under vacuum at 60°C before embedding in Taab embedding capsules and polymerization of the resin at 60°C for 48 h. Survey sections of 0.5 μm thickness were stained with toluidine blue to identify areas of optimal cell density. Ultrathin sections (60 nm) were then prepared using a Diatome diamond knife on a Leica UC6 ultramicrotome and stained with uranyl acetate and lead citrate for examination with a Philips CM10 transmission microscope (FEI UK Ltd., Cambridge, United Kingdom) and imaging with a Gatan BioScan 792 (Gatan UK, Abingdon, United Kingdom).

### Wheat germ agglutinin-colloidal gold staining of cell wall chitin.

To examine chitin distribution in cell walls, transmission electron microscopy (TEM) thin sections were stained with the lectin wheat germ agglutinin (WGA), which was conjugated to colloidal gold particles (34–36). Unstained ultrathin sections were mounted on 300-mesh nickel grids (Agar Scientific Ltd., Essex, United Kingdom) and labeled with 10 nm WGA-colloidal gold particles (British Biocell International Ltd., Cardiff, United Kingdom). All incubation steps were performed at room temperature by placing the grids into drops of reagent on dental wax. The grids were immersed for 1 h in WGA-gold which had been diluted 1:5 with Tris-buffered saline (TBS). Nonspecific binding of colloidal gold was tested with a 1:10 dilution of goat anti-mouse F(ab′)_2_ conjugated to colloidal gold (British Biocell International Ltd., Cardiff, United Kingdom). All grids were washed with drops and jet rinsing in TBS followed by distilled water (dH_2_O). Thin sections were poststained for 10 min with 5% (wt/vol) aqueous uranyl acetate and with lead citrate for 4 min (37).

### Time-lapse observations using microfluidics.

The ONIX microfluidic perfusion system (CellASIC Corp., USA) was used to analyze the dynamic responses of A. fumigatus cells when perfused with caspofungin-supplemented medium. According to the manufacturer's instructions, the flow rate and exchanging solutions were controlled by pressure (pounds per square inch [psi]) using the ONIX FG software v2.6. Spores were diluted to ∼5 × 10^4^ in Sabdex broth containing 5 μg/ml CFW to visualize chitin and applied to a microfluidics plate, Y04C (CellASIC Corp., USA). Approximately 50 spores were loaded into each chamber. Sabdex broth containing CaCl_2_ and CFW was consistently perfused through the chamber with a flow rate of 4 lb/in^2^ (∼10 μl/h) for 6 h. Then cells were treated with 32 μg/ml caspofungin for a further 6 h and grown in fresh medium for 8 h. Cells in the chamber were observed using a DeltaVision Core microscope (Image Solutions Ltd., Preston, United Kingdom). All images were taken using a CoolSNAP camera (Photometrics UK Ltd., London, United Kingdom). Image analysis was performed using ImageJ v1.45 free software (http://rsbweb.nih.gov/ij/).

## RESULTS

### Morphological changes in A. fumigatus hyphae in response to caspofungin treatment.

**FIG 1:**
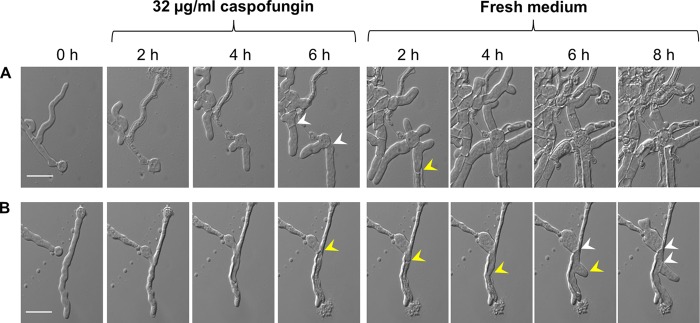
Morphological changes in A. fumigatus in response to caspofungin. A. fumigatus spores were trapped in a microfluidics chamber and grown in Sabouraud plus 2% glucose for 6 h in the absence (A) or presence (B) of CaCl_2_ and CFW. Then all cells were treated with 32 μg/ml caspofungin for another 6 h and grown in fresh medium for 8 h. Yellow arrowheads indicate intrahyphal hyphae. White arrowheads indicate newly formed septum. Scale bars = 20 μm.

The effect of caspofungin on the morphology of single germlings of A. fumigatus that had been pretreated with CaCl_2_ and CFW (Fig. 1B) compared to its effect on sham-treated controls was examined in real time using a microfluidics system (Fig. 1A). Exposure of A. fumigatus to 32 μg/ml caspofungin, after pregrowth with or without CaCl_2_ and CFW, resulted in bursting of hyphal tips (Fig. 1). After lysis of hyphal tips, incidences of septum formation distal to the burst hyphal apices were observed (Fig. 1). Removal of caspofungin from the growth medium resulted in new apical growth of some of the burst hyphae (Fig. 1). The microfluidics chambers were then perfused with medium containing no drug. After 6 to 8 h, there was evidence of septum formation and of intrahyphal growth within dead cells (Fig. 1). Therefore, caspofungin induced tip lysis but did not sterilize cultures and sporadic septation and hyphal growth resumed in the continued presence of caspofungin.

### Caspofungin treatment induces a compensatory increase in chitin content in A. fumigatus.

**FIG 2:**
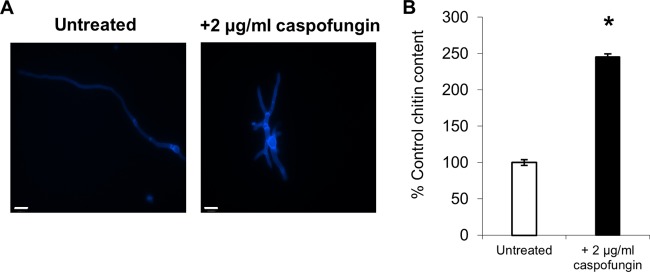
Treatment with caspofungin leads to a compensatory increase in chitin content in A. fumigatus. The wild-type strain was grown at 37°C for 12 h in RPMI 1640 in the presence and absence of 2 μg/ml caspofungin. (A) CFW-stained fluorescent images; scale bars are 10 μm. (B) Cell wall chitin assays were performed three times on three biologically independent samples (average ± standard deviation [SD], *n* = 9). The asterisk indicates significant difference (*P* < 0.05) from untreated cells.

**FIG 3:**
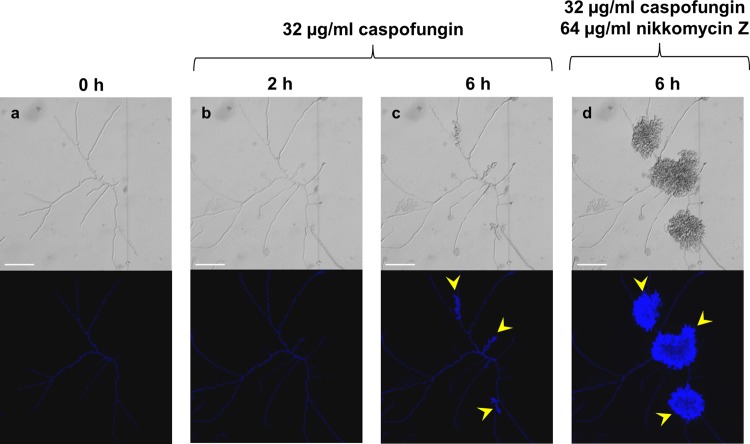
Intrahyphal growth of A. fumigatus corresponds to areas of increased chitin content in response to caspofungin treatment. A. fumigatus spores were trapped in a microfluidics chamber and grown in Sabdex broth plus 2% glucose for 2 h. Cells were then treated with 32 μg/ml caspofungin for 6 h, followed by combination treatment with 32 μg/ml caspofungin and 64 μg/ml nikkomycin Z for a further 6 h. Yellow arrowheads indicate intrahyphal hyphae. DIC (top) and CFW fluorescent images (bottom). Scale bars = 10 μm.

A. fumigatus conidia were germinated for 12 h in RPMI 1640 with and without caspofungin. Chitin levels were determined by staining hyphae with CFW and by measuring cell wall chitin content by high-pressure liquid chromatography (HPLC). Staining with CFW showed an increase in chitin in A. fumigatus hyphae after treatment with caspofungin compared to untreated controls (Fig. 2A), as reported previously (38, 39). Treatment of A. fumigatus with caspofungin resulted in the formation of short, stumpy, hyperbranched hyphae (Fig. 2A). Biochemical measurement of chitin content demonstrated that hyphae treated with 2 μg/ml caspofungin had a 2.5-fold increase in their cell wall chitin compared to untreated controls (Fig. 2B). The increase in chitin content of A. fumigatus germlings in response to caspofungin treatment was also examined in real time using a microfluidics perfusion system (Fig. 3). Treatment with caspofungin led to an initial lysis of most of the hyphal tips (Fig. 3b). However, following lysis of hyphal tips, an increase in chitin content within some hyphal regions distal to the sites of tip lysis was observed that was coincident with the resumption of growth of wide, hyperbranched hyphae (Fig. 3c). In some experiments, cultures exhibiting this adapted growth phenotype were perfused with 64 μg/ml nikkomycin Z in combination with caspofungin, but this did not inhibit growth (Fig. 3d).

**FIG 4:**
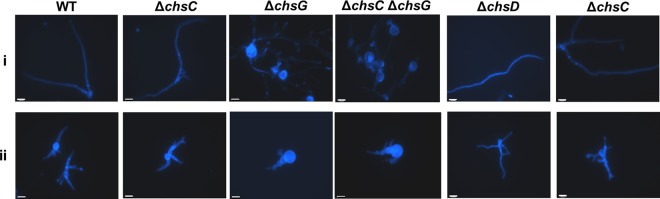
*AfCHSG* is required for the compensatory increase in chitin content in response to caspofungin treatment. CFW-stained fluorescent images of the wild-type (WT) strain and Δ*Afchs* mutants in RPMI 1640 alone (i) or supplemented with 2 μg/ml caspofungin (ii) after 12 h at 37°C. Scale bars = 10 μm.

Next, a series of Δ*Afchs* disruption mutants was stained with CFW to establish which *CHS* enzymes were required for the compensatory increase in chitin synthesis induced by exposure to caspofungin (Fig. 4i). Most untreated Δ*Afchs* mutants had similar chitin content to the wild type, with the exception of the Δ*Afchs*G mutant, which exhibited swollen cells with high CFW staining (Fig. 4i). Treatment with caspofungin resulted in a compensatory increase in chitin content in the individual Δ*Afchs* mutants compared to untreated strains, with the exception of strains lacking *AfCHSG* (Fig. 4ii).

### The class III chitin synthase, encoded by *AfCHSG*, is involved in the synergistic action of caspofungin and nikkomycin Z.

**FIG 5:**
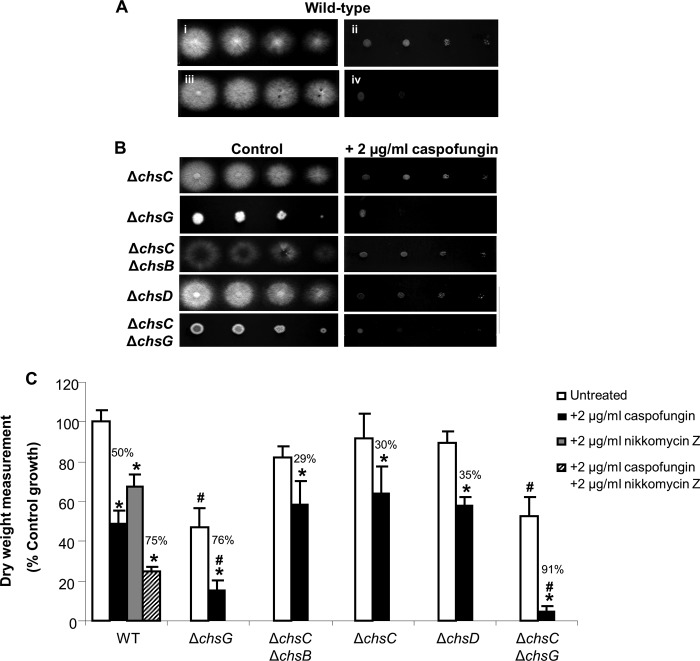
Disruption of *AfCHSG* leads to hypersensitivity to caspofungin. (A) Plate dilution sensitivity tests of the A. fumigatus wild-type strain on RPMI 1640 agar alone (i) or supplemented with 2 μg/ml caspofungin (ii), 2 μg/ml nikkomycin Z (iii), or a combination (iv). (B) The Δ*Afchs* mutants were grown on RPMI 1640 agar with or without 2 μg/ml caspofungin. Plates were incubated for 48 h at 37°C. Spore numbers per spot are 5,000, 500, 50, and 5 spores, from left to right. (C) Dry weights of wild-type and Δ*Afchs* mutants were determined after 24 h growth in RPMI 1640 broth alone or supplemented with 2 μg/ml caspofungin. The wild-type strain was also treated with 2 μg/ml nikkomycin Z alone and in combination with caspofungin. Error bars are SD (*n* = 3, from three independent experiments). Asterisks indicate significant differences (*P* < 0.05) from untreated cells of the same genetic background. #, significant difference (*P* < 0.05) from the wild-type cells in the same growth conditions. Numbers represent percentages of growth inhibition compared to untreated cells of the same genetic background.

Caspofungin has a fungistatic effect on the growth of A. fumigatus and results in a compensatory increase in chitin synthesis (Fig. 2). Since chitin upregulation affected caspofungin efficacy, we assessed whether combinations of chitin synthase inhibitors and caspofungin acted synergistically. The addition of 2 μg/ml of the chitin synthase inhibitor nikkomycin Z did not affect growth on RPMI 1640 agar, whereas the addition of 2 μg/ml caspofungin led to a dramatic reduction in colony size (Fig. 5A). Combined treatment with caspofungin and nikkomycin Z resulted in the formation of colonies with substantially reduced radial growth compared to treatment with caspofungin alone (Fig. 5A).

The sensitivity of the Δ*Afchs* mutants to caspofungin was determined. All of the Δ*Afchs* mutants grew similarly to the wild type on RPMI 1640 alone, with the exception of the Δ*AfchsG* and Δ*AfchsC* Δ*AfchsG* mutants, which were viable but had reduced radial growth (Fig. 5B). Similarly, treatment with caspofungin led to a reduction in colony diameter, and the inhibition of growth was similar to wild-type cells for the majority of Δ*Afchs* mutants (Fig. 5B). Exceptions were the Δ*AfchsG* and Δ*AfchsC* Δ*AfchsG* mutants, which were hypersensitive to caspofungin (Fig. 5B). Treatment with 2 μg/ml caspofungin alone led to a 50% reduction in growth of wild-type cells, whereas treatment with 2 μg/ml nikkomycin Z alone led to a 30% reduction in growth (Fig. 5C). Combined treatment of caspofungin and nikkomycin Z at the same concentrations had an additive effect and resulted in a 75% reduction in growth of wild-type cells (Fig. 5C). When grown in RPMI 1640 alone, all Δ*Afchs* mutants exhibited growth that was comparable to the wild type, again with the exception of the Δ*AfchsG* and Δ*AfchsC* Δ*AfchsG* mutants, which had a 45% reduction in growth (Fig. 5C). Similarly, treatment of the Δ*Afchs* mutants with caspofungin resulted in a 35% reduction in growth that was comparable to that of the wild type. Again the exception was the Δ*AfchsG* and Δ*AfchsC* Δ*AfchsG* mutants, which demonstrated an 80% to 90% reduction in growth in the presence of caspofungin (Fig. 5C). The reduction in growth of the Δ*AfchsG* and Δ*AfchsC* Δ*AfchsG* mutants in the presence of caspofungin was comparable to the reduction in growth observed when wild-type cells were treated with a combination of caspofungin and nikkomycin Z (Fig. 5C). Therefore, the class III chitin synthase *Af*ChsG was most critically involved in the response to caspofungin treatment.

### Treatment with CaCl_2_ and CFW increases chitin content in A. fumigatus.

**FIG 6:**
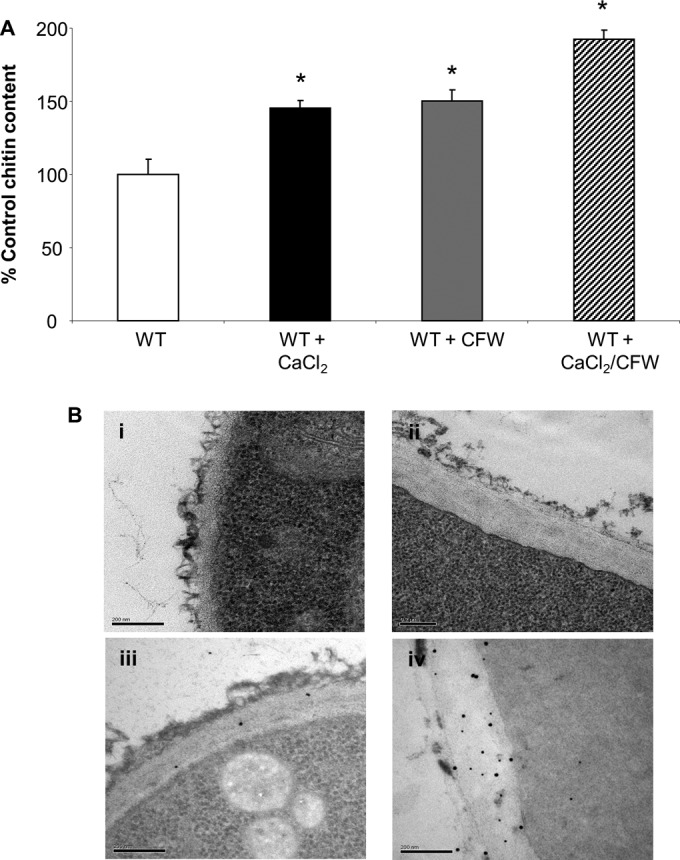
Treatment with CaCl_2_ and CFW increases A. fumigatus cell wall chitin. The A. fumigatus wild-type strain (H237) was grown at 37°C for 8 h in Sabouraud dextrose broth alone and supplemented with 200 mM CaCl_2_ or 100 μg/ml CFW or a combination. (A) Cell wall chitin assays were performed three times on three biologically independent samples (average ± SD, *n* = 9). An asterisk indicates significant difference (*P* < 0.05) from untreated cells. (B) TEM images of the wild-type strain grown at 37°C for 8 h in Sabouraud dextrose broth alone (i) and supplemented with 200 mM CaCl_2_ and 100 μg/ml CFW (ii). WGA-colloidal gold-stained TEM sections showing chitin in untreated wild-type cells (iii) and wild-type cells treated with CaCl_2_ and CFW (iv). Scale bars = 0.2 μm.

Previously, treatment with CaCl_2_ and CFW was shown to increase the chitin content of C. albicans and reduce susceptibility to caspofungin (19, 23). To determine whether treatment with CaCl_2_ and CFW also increased the chitin content of A. fumigatus, spores were germinated in Sabdex broth with and without a combination of 200 mM CaCl_2_ and 100 μg/ml CFW for 8 h. Hyphae that had germinated in the presence of CaCl_2_ and CFW had a 2-fold increase in cell wall chitin content, measured by HPLC, compared to that of the wild type (Fig. 6A). Ultrastructural analysis using transmission electron microscopy revealed that CaCl_2_- and CFW-treated hyphae had 45% thicker cell walls (Fig. 6Bii), which were chitin rich (Fig. 6Biv) relative to those of untreated hyphae (Fig. 6Bi and Biii).

**FIG 7:**
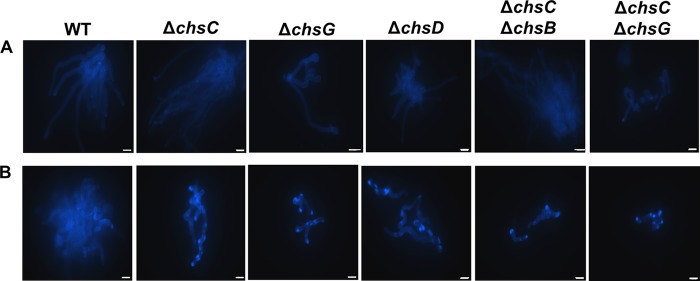
Treatment with CaCl_2_ and CFW leads to an increase in chitin synthesis in Δ*Afchs* mutants of A. fumigatus. CFW-stained fluorescent images of the wild-type strain and Δ*Afchs* mutants in Sabdex broth alone (A) or supplemented with 200 mM CaCl_2_ and 100 μg/ml CFW (B) after 12 h at 37°C. Scale bars = 10 μm.

Δ*Afchs* mutants were also treated with the CaCl_2_ and CFW combinations, and their relative chitin contents were determined by staining with 25 μg/ml CFW. Treatment with CaCl_2_ and CFW led to defined chitin-rich patches in all Δ*Afchs* mutants at some hyphal tips and at the kinks and bends of hyphal cells. The CFW-rich patches were evident in multiple Δ*Afchs* null mutants, suggesting that no single *CHS* was responsible exclusively for the increase in chitin content resulting from caspofungin exposure (Fig. 7). However, in the wild-type strain, there was a more uniform increase in chitin staining along the hypha, perhaps indicating that some differences exist between the mutants and the wild type in the spatial deposition of chitin upon CaCl_2_ and CFW cotreatment.

### Activation of the cell wall salvage pathway protects against caspofungin treatment.

**FIG 8:**
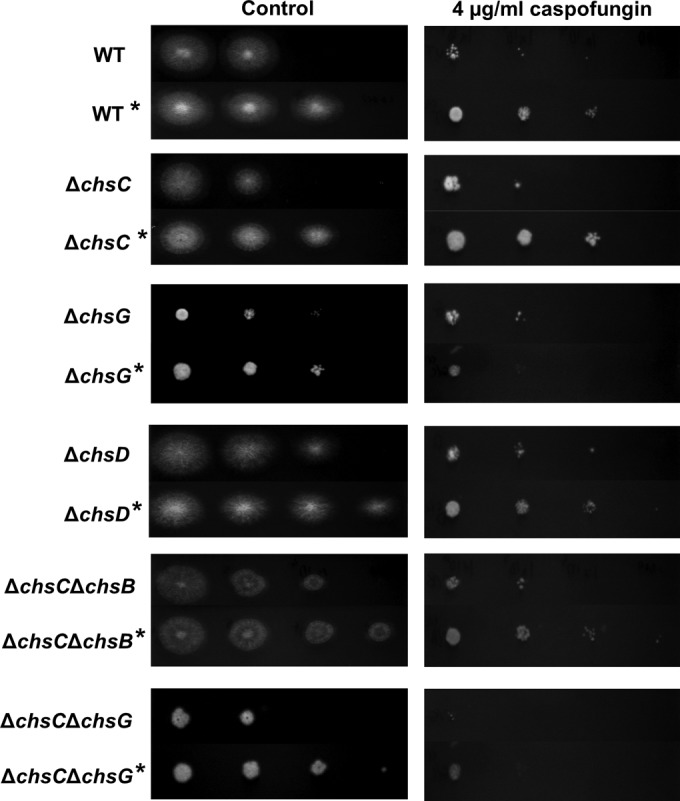
Pregrowing A. fumigatus in CaCl_2_ and CFW reduces susceptibility to caspofungin on solid medium. Plate dilution sensitivity tests of the A. fumigatus wild-type strain (H237) and various Δ*Afchs* mutants on RPMI 1640 agar supplemented with 4 μg/ml caspofungin. Rows marked with an asterisk indicate pregrowth of the inoculum in Sabdex broth containing 200 mM CaCl_2_ and 100 μg/ml CFW. Plates were incubated for 24 h at 37°C. Spore numbers per spot are 5,000, 500, 50, and 5 spores, from left to right.

To determine whether increased chitin content leads to reduced caspofungin susceptibility in A. fumigatus, conidia were first germinated in Sabdex broth with and without 200 mM CaCl_2_ and 100 μg/ml CFW to elevate chitin, then hyphae were washed and exposed to caspofungin. Pregrowth with CaCl_2_ and CFW enhanced the growth of the wild type and Δ*Afchs* mutants on control plates and led to reduced susceptibility to caspofungin in all strains tested, with the exception of strains lacking *AfCHSG* (Fig. 8). When strains lacking *AfCHSG* were grown with CaCl_2_ and CFW prior to caspofungin treatment, the radial growth of their colonies was still reduced compared to that of the colonies grown without CaCl_2_ and CFW pretreatment (Fig. 8).

The caspofungin MECs for untreated and pretreated strains were measured in liquid RPMI 1640. In most cases, pregrowth with CaCl_2_ and CFW led to a significant reduction in susceptibility to caspofungin (Table 2). Again, the exceptions were the Δ*AfchsG* and Δ*AfchsC* Δ*AfchsG* mutants, which retained their increased susceptibility to caspofungin even after pregrowth with CaCl_2_ and CFW. Therefore, *AfCHSG* was critical for the protective upregulation of chitin synthesis in A. fumigatus.

**TABLE 2:** Pretreatment with CaCl_2_ and CFW increases the caspofungin MEC against A. fumigatus strains

A. fumigatus strain description	MEC (μg/ml)	
	Untreated	Pretreated (CaCl_2_ and CFW)
Wild type	0.5	4
Δ*chsC*	0.5	4
Δ*chsG*	0.064	0.064
Δ*chsD*	0.25	8
Δ*chsC* Δ*chsB*	0.25	4
Δ*chsC* Δ*chsG*	0.064	0.064

## DISCUSSION

We show here that exposure to the echinocandin caspofungin can lead to the upregulation of chitin synthesis mediated by *Af*ChsG and subsequent survival and growth of aberrant chitin-rich hyperbranched hyphae. The importance of these observations is that this chitin-rich surviving biomass has the potential to act as a reservoir for regrowth of Aspergillus mycelium following echinocandin treatment.

Different species of Aspergillus have various susceptibilities to the echinocandins, and several examples of echinocandin resistance in Aspergillus species have been reported (17, 40–48). Anidulafungin displays the greatest inhibition of growth across the Aspergillus spp. compared to that of caspofungin and micafungin. Generally, clinical isolates of A. fumigatus, A. terreus, and A. flavus have been reported to have comparable susceptibilities to all three echinocandins (43). In contrast, A. niger has been shown to be considerably more susceptible to caspofungin (MEC, 0.1 to 0.5 μg/ml) than A. fumigatus (MEC, 0.2 to 6 μg/ml), which is thought to be due to differences in cell wall composition (17). Potential mechanisms of resistance to caspofungin in A. fumigatus have been highlighted by two classes of laboratory-generated mutants that have reduced susceptibility to caspofungin (49). Point mutations within *AfFKS1* leading to an S678P amino acid substitution resulted in an MEC of 4 μg/ml, compared to an MEC of 0.25 μg/ml with the susceptible wild-type strain (49, 50). This was considered a low level of resistance, as the MEC was only 16-fold higher. In contrast, spontaneous caspofungin-resistant mutants that were generated by cell wall digestion, followed by regeneration of spheroplasts on caspofungin-containing medium, displayed higher levels of resistance (49). Expression profiling of these mutants after treatment with caspofungin showed upregulation of genes involved in cell wall biosynthesis/remodelling, structural cell components, and transport (49). In addition, a clinical isolate of A. fumigatus from a patient who failed caspofungin therapy was shown to be resistant due to overexpression of the *AfFKS1* gene (51). Two recent studies have also demonstrated that clinical resistance to caspofungin in Aspergillus spp. occurred at a frequency of 4% to 6% in cancer patients and transplant recipients (40, 45). Furthermore, there have been several reports of the emergence of anidulafungin-resistant molds in the clinic (40, 42, 44, 45).

In C. albicans and A. fumigatus, growth at high concentrations above the MIC of caspofungin, termed paradoxical growth, has been observed *in vitro*. Paradoxical growth occurs most commonly when A. fumigatus is treated with caspofungin, rather than micafungin or anidulafungin, *in vitro* (38, 43). In C. albicans, cells demonstrating paradoxical growth were shown to have a 900% increase in chitin content, suggesting that the regrowth at high concentrations of caspofungin was due to an increase in chitin content (52). In A. fumigatus, addition of the calcineurin inhibitor, FK506, or deletion of genes from the calcineurin pathway abolished the occurrence of paradoxical growth (38). Rogg et al. (53) demonstrated that the Ca^2+^-calcineurin pathway was required for the transcriptional upregulation of *AfCHSA* and *AfCHSC* in response to caspofungin treatment. Likewise, chitin content and chitin synthase enzyme activity were also increased during paradoxical growth (38). A likely hypothesis is therefore that paradoxical effects arise because high levels of echinocandins are able to activate the cell wall salvage pathway(s) that promote chitin synthesis and cell survival.

The data presented here highlight the potential of increased chitin content as a mechanism of reduced susceptibility to caspofungin in A. fumigatus. Treatment of wild-type cells with caspofungin resulted in a 2.5-fold compensatory increase in chitin content. This supports the findings of previous studies where a different wild-type strain of A. fumigatus also demonstrated a compensatory increase in chitin content in response to treatment with all three echinocandins (21, 39). Here we show that disruption of the class II, III, and VI chitin synthase genes did not markedly affect the ability of A. fumigatus to increase chitin synthesis to compensate for the inhibition of β-1,3-glucan synthesis by caspofungin. Therefore, the remaining *AfCHS* genes may be deduced to be able to compensate for the loss of the class II, III, and VI chitin synthase enzymes by synthesizing sufficient chitin to protect the fungus from caspofungin. Supporting this, combined treatments of A. fumigatus with caspofungin and nikkomycin Z did not inhibit the compensatory increase in chitin content (21). The compensatory increase in chitin content of A. fumigatus in response to caspofungin treatment has also been shown to be dependent on the A. fumigatus Ca^2+^-calcineurin pathway genes *AfCNAA* and *AfCRZA* (21).

In C. albicans, pregrowth of cells with a combination of CaCl_2_ and CFW activates the Ca^2+^-calcineurin and PKC signaling pathways, resulting in a 3- to 4-fold increase in chitin content (23). This elevation of chitin content renders C. albicans cells less susceptible to caspofungin (19). Pregrowth of A. fumigatus with CaCl_2_ and CFW also resulted in an increase in chitin content, which led to reduced susceptibility to caspofungin. This increase in chitin content was not solely dependent on the class II, III, and VI chitin synthase enzymes because mutants lacking enzymes from these three classes still demonstrated an increase in chitin content. Despite this, the Δ*AfchsG* and the Δ*AfchsC* Δ*AfchsG* mutants had no decrease in caspofungin susceptibility after treatment with CaCl_2_ and CFW. Our results suggest that *AfCHSG* makes a major contribution to the ability of CaCl_2_- and CFW-treated cells to grow in the presence of caspofungin. Mutants lacking *AfCHSG* may grow in 4 μg/ml caspofungin after CaCl_2_ and CFW treatment compared to the other strains tested. The other Chs enzymes are therefore likely to make some contribution to the cell wall salvage mechanism, primed by CaCl_2_ and CFW treatment, because mutants lacking *AfCHSG* still upregulated chitin production, even though this was not sufficient to decrease sensitivity to caspofungin. Therefore, increasing the cell wall chitin content is also a mechanism of tolerance to caspofungin in filamentous fungi such as A. fumigatus.

Here we observed that treatment with caspofungin promoted intrahyphal growth within lysed hyphae, which may contribute to the ability of A. fumigatus to survive caspofungin treatment. In other filamentous fungi, intrahyphal hyphae have been proposed to promote fungal survival in response to stress conditions (54). Supporting this, recent work has shown that compounds which inhibit septum formation act synergistically with caspofungin (39). However, this is the first example of the formation of intrahyphal hyphae as a response to echinocandin treatment.

A. fumigatus activates a compensatory increase in chitin content in response to sub-MIC caspofungin treatment, which highlights the potential of combining chitin synthase inhibitors with the echinocandins for improved and/or broader spectrum therapy. In addition, the PKC, Ca^2+^-calcineurin, and HOG signaling pathways have been shown to be required for the response of C. albicans and A. fumigatus to caspofungin and the *in vitro* paradoxical growth phenomenon (19–21, 55, 56). Consequently, inhibitors of these pathways together with an echinocandin should be explored as possible combination therapies.

Chitin synthase inhibitors have been shown to enhance the activity of caspofungin and other echinocandins against a range of fungal pathogens (19, 57–60). Combination treatment with chitin inhibitors and the echinocandins may, therefore, add potency and increase the spectrum of activity of echinocandins to a range of filamentous fungal pathogens. For example, synergistic inhibition of Alternaria infectoria with caspofungin and nikkomycin Z has been reported (61). Combination treatment of A. fumigatus with nikkomycin Z and the echinocandins leads to enhanced killing *in vitro* and results in the formation of swollen spores with aberrant walls, which are prone to lysis (21, 58, 62). Also, treatment with micafungin significantly prolonged host survival in systemic and pulmonary murine aspergillosis when combined with nikkomycin Z (63, 64).

This study illuminates the conserved clinical relevance of the cell wall compensatory mechanism in response to assaults that weaken the wall. A common feature of this mechanism is the activation of chitin synthesis, which involves multiple members of fungal chitin synthase families. These pathways are activated when fungi are exposed to sub-MIC echinocandins and may contribute to tolerance if drugs are administered suboptimally or there is reduced bioavailability. The ability to simultaneously block the synthesis of chitin and β-1,3-glucan represents a major opportunity for future strategies to augment the range and cidality of echinocandins.

## Supplementary Material

Supplemental material

## References

[1] Brown GD, Denning DW, Gow NA, Levitz SM, Netea MG, White TC 2012 Hidden killers: human fungal infections. Sci Transl Med 4:165rv13. doi:10.1126/scitranslmed.3004404. 23253612

[2] Mellado E, Aufauvre-Brown A, Specht CA, Robbins PW, Holden DW 1995 A multigene family related to chitin synthase genes of yeast in the opportunistic pathogen Aspergillus fumigatus. Mol Gen Genet 246:353–359. doi:10.1007/BF00288608. 7854320

[3] Latge JP 2007 The cell wall: a carbohydrate armour for the fungal cell. Mol Microbiol 66:279–290. doi:10.1111/j.1365-2958.2007.05872.x. 17854405

[4] Latge JP 2001 The pathobiology of Aspergillus fumigatus. Trends Microbiol 9:382–389. doi:10.1016/S0966-842X(01)02104-7. 11514221

[5] Alcazar-Fuoli L, Mellado E, Alastruey-Izquierdo A, Cuenca-Estrella M, Rodriguez-Tudela JL 2008 Aspergillus section Fumigati: antifungal susceptibility patterns and sequence-based identification. Antimicrob Agents Chemother 52:1244–1251. doi:10.1128/AAC.00942-07. 18212093 PMC2292508

[6] van der Linden JW, Snelders E, Kampinga GA, Rijnders BJ, Mattsson E, Debets-Ossenkopp YJ, Kuijper EJ, Van Tiel FH, Melchers WJ, Verweij PE 2011 Clinical implications of azole resistance in Aspergillus fumigatus, The Netherlands, 2007-2009. Emerg Infect Dis 17:1846–1854. doi:10.3201/eid1710.110226. 22000354 PMC3311118

[7] Lionakis MS, Lewis RE, Torres HA, Albert ND, Raad II, Kontoyiannis DP 2005 Increased frequency of non-fumigatus Aspergillus species in amphotericin B- or triazole-pre-exposed cancer patients with positive cultures for aspergilli. Diagn Microbiol Infect Dis 52:15–20. doi:10.1016/j.diagmicrobio.2005.01.001. 15878437

[8] Perkhofer S, Lass-Florl C, Hell M, Russ G, Krause R, Honigl M, Geltner C, Auberger J, Gastl G, Mitterbauer M, Willinger B, Knobl P, Resch G, Waldner R, Makrai A, Hartmann G, Girschikofsky M, Greil R 2010 The Nationwide Austrian Aspergillus Registry: a prospective data collection on epidemiology, therapy and outcome of invasive mould infections in immunocompromised and/or immunosuppressed patients. Int J Antimicrob Agents 36:531–536. doi:10.1016/j.ijantimicag.2010.08.010. 20947312

[9] Verweij PE, Snelders E, Kema GH, Mellado E, Melchers WJ 2009 Azole resistance in Aspergillus fumigatus: a side-effect of environmental fungicide use? Lancet Infect Dis 9:789–795. doi:10.1016/S1473-3099(09)70265-8. 19926038

[10] Howard SJ, Harrison E, Bowyer P, Varga J, Denning DW 2011 Cryptic species and azole resistance in the Aspergillus niger complex. Antimicrob Agents Chemother 55:4802–4809. doi:10.1128/AAC.00304-11. 21768508 PMC3186969

[11] Epaulard O, Villier C, Ravaud P, Chosidow O, Blanche S, Mamzer-Bruneel MF, Thiebaut A, Leccia MT, Lortholary O 2013 A multistep voriconazole-related phototoxic pathway may lead to skin carcinoma: results from a French nationwide study. Clin Infect Dis 57:e182–e188. doi:10.1093/cid/cit600. 24046296

[12] Bowman JC, Hicks PS, Kurtz MB, Rosen H, Schmatz DM, Liberator PA, Douglas CM 2002 The antifungal echinocandin caspofungin acetate kills growing cells of Aspergillus fumigatus *in vitro*. Antimicrob Agents Chemother 46:3001–3012. doi:10.1128/AAC.46.9.3001-3012.2002. 12183260 PMC127409

[13] Odds FC, Brown AJ, Gow NA 2003 Antifungal agents: mechanisms of action. Trends Microbiol 11:272–279. doi:10.1016/S0966-842X(03)00117-3. 12823944

[14] Kurtz MB, Heath IB, Marrinan J, Dreikorn S, Onishi J, Douglas C 1994 Morphological effects of lipopeptides against Aspergillus fumigatus correlate with activities against (1,3)-beta-d-glucan synthase. Antimicrob Agents Chemother 38:1480–1489. doi:10.1128/AAC.38.7.1480. 7979276 PMC284580

[15] Arikan S, Lozano-Chiu M, Paetznick V, Rex JH 2001 *In vitro* susceptibility testing methods for caspofungin against Aspergillus and Fusarium isolates. Antimicrob Agents Chemother 45:327–330. doi:10.1128/AAC.45.1.327-330.2001. 11120990 PMC90285

[16] Espinel-Ingroff A 2003 *In vitro* antifungal activities of anidulafungin and micafungin, licensed agents and the investigational triazole posaconazole as determined by NCCLS methods for 12,052 fungal isolates: review of the literature. Rev Iberoam Micol 20:121–136. 15456349

[17] Imhof A, Balajee SA, Marr KA 2003 New methods to assess susceptibilities of Aspergillus isolates to caspofungin. J Clin Microbiol 41:5683–5688. doi:10.1128/JCM.41.12.5683-5688.2003. 14662961 PMC309030

[18] Beauvais A, Bruneau JM, Mol PC, Buitrago MJ, Legrand R, Latge JP 2001 Glucan synthase complex of Aspergillus fumigatus. J Bacteriol 183:2273–2279. doi:10.1128/JB.183.7.2273-2279.2001. 11244067 PMC95134

[19] Walker LA, Munro CA, de Bruijn I, Lenardon MD, McKinnon A, Gow NA 2008 Stimulation of chitin synthesis rescues Candida albicans from echinocandins. PLoS Pathog 4:e1000040. doi:10.1371/journal.ppat.1000040. 18389063 PMC2271054

[20] Cota JM, Grabinski JL, Talbert RL, Burgess DS, Rogers PD, Edlind TD, Wiederhold NP 2008 Increases in *SLT2* expression and chitin content are associated with incomplete killing of Candida glabrata by caspofungin. Antimicrob Agents Chemother 52:1144–1146. doi:10.1128/AAC.01542-07. 18086838 PMC2258485

[21] Fortwendel JR, Juvvadi PR, Pinchai N, Perfect BZ, Alspaugh JA, Perfect JR, Steinbach WJ 2009 Differential effects of inhibiting chitin and 1,3-β-d-glucan synthesis in ras and calcineurin mutants of Aspergillus fumigatus. Antimicrob Agents Chemother 53:476–482. doi:10.1128/AAC.01154-08. 19015336 PMC2630655

[22] Walker LA, Gow NA, Munro CA 2013 Elevated chitin content reduces the susceptibility of Candida species to caspofungin. Antimicrob Agents Chemother 57:146–154. doi:10.1128/AAC.01486-12. 23089748 PMC3535899

[23] Munro CA, Selvaggini S, de Bruijn I, Walker L, Lenardon MD, Gerssen B, Milne S, Brown AJ, Gow NA 2007 The PKC, HOG and Ca^2+^ signalling pathways co-ordinately regulate chitin synthesis in Candida albicans. Mol Microbiol 63:1399–1413. doi:10.1111/j.1365-2958.2007.05588.x. 17302816 PMC2649417

[24] Mora-Montes HM, Netea MG, Ferwerda G, Lenardon MD, Brown GD, Mistry AR, Kullberg BJ, O'Callaghan CA, Sheth CC, Odds FC, Brown AJ, Munro CA, Gow NA 2011 Recognition and blocking of innate immunity cells by Candida albicans chitin. Infect Immun 79:1961–1970. doi:10.1128/IAI.01282-10. 21357722 PMC3088140

[25] Lee KK, Maccallum DM, Jacobsen MD, Walker LA, Odds FC, Gow NA, Munro CA 2012 Elevated cell wall chitin in Candida albicans confers echinocandin resistance *in vivo*. Antimicrob Agents Chemother 56:208–217. doi:10.1128/AAC.00683-11. 21986821 PMC3256049

[26] Mellado E, Aufauvre-Brown A, Gow NA, Holden DW 1996 The Aspergillus fumigatus *chsC* and *chsG* genes encode class III chitin synthases with different functions. Mol Microbiol 20:667–679. doi:10.1046/j.1365-2958.1996.5571084.x. 8736545

[27] Mellado E, Specht CA, Robbins PW, Holden DW 1996 Cloning and characterization of *chsD*, a chitin synthase-like gene of Aspergillus fumigatus. FEMS Microbiol Lett 143:69–76. doi:10.1111/j.1574-6968.1996.tb08463.x. 8807804

[28] Jimenez-Ortigosa C, Aimanianda V, Muszkieta L, Mouyna I, Alsteens D, Pire S, Beau R, Krappmann S, Beauvais A, Dufrene YF, Roncero C, Latge JP 2012 Chitin synthases with a myosin motor-like domain control the resistance of Aspergillus fumigatus to echinocandins. Antimicrob Agents Chemother 56:6121–6131. doi:10.1128/AAC.00752-12. 22964252 PMC3497188

[29] Muszkieta L, Aimanianda V, Mellado E, Gribaldo S, Alcazar-Fuoli L, Szewczyk E, Prevost MC, Latge JP 2014 Deciphering the role of the chitin synthase families 1 and 2 in the *in vivo* and *in vitro* growth of Aspergillus fumigatus by multiple gene targeting deletion. Cell Microbiol 16:1784–1805. doi:10.1111/cmi.12326. 24946720

[30] Borgia PT, Iartchouk N, Riggle PJ, Winter KR, Koltin Y, Bulawa CE 1996 The *chsB* gene of Aspergillus nidulans is necessary for normal hyphal growth and development. Fungal Genet Biol 20:193–203. doi:10.1006/fgbi.1996.0035. 8953267

[31] Mellado E, Dubreucq G, Mol P, Sarfati J, Paris S, Diaquin M, Holden DW, Rodriguez-Tudela JL, Latge JP 2003 Cell wall biogenesis in a double chitin synthase mutant (*chsG*-/*chsE*-) of Aspergillus fumigatus. Fungal Genet Biol 38:98–109. doi:10.1016/S1087-1845(02)00516-9. 12553940

[32] Aufauvre-Brown A, Mellado E, Gow NAR, Holden DW 1997 Aspergillus fumigatus *chsE*: a gene related to *CHS3* of Saccharomyces cerevisiae and important for hyphal growth and conidiophore development but not pathogenicity. Fungal Genet Biol 21:141–152. doi:10.1006/fgbi.1997.0959. 9126623

[33] Clinical and Laboratory Standards Institute. 2008 Reference method for broth dilution antifungal susceptibility testing of filamentous fungi; approved standard—2nd ed CLSI document M38-A2. Clinical and Laboratory Standards Institute, Wayne, PA.

[34] Hilenski LL, Naider F, Becker JM 1986 Polyoxin D inhibits colloidal gold-wheat germ agglutinin labelling of chitin in dimorphic forms of Candida albicans. J Gen Microbiol 132:1441–1451. 3543207 10.1099/00221287-132-6-1441

[35] Munro CA, Winter K, Buchan A, Henry K, Becker JM, Brown AJ, Bulawa CE, Gow NA 2001 Chs1 of Candida albicans is an essential chitin synthase required for synthesis of the septum and for cell integrity. Mol Microbiol 39:1414–1426. 11251855 10.1046/j.1365-2958.2001.02347.x

[36] Tronchin G, Poulain D, Herbaut J, Biguet J 1981 Localization of chitin in the cell wall of Candida albicans by means of wheat germ agglutinin. Fluorescence and ultrastructural studies. Eur J Cell Biol 26:121–128. 7035173

[37] Reynolds ES 1963 The use of lead citrate at high pH as an electron-opaque stain in electron microscopy. J Cell Biol 17:208–212. doi:10.1083/jcb.17.1.208. 13986422 PMC2106263

[38] Fortwendel JR, Juvvadi PR, Perfect BZ, Rogg LE, Perfect JR, Steinbach WJ 2010 Transcriptional regulation of chitin synthases by calcineurin controls paradoxical growth of Aspergillus fumigatus in response to caspofungin. Antimicrob Agents Chemother 54:1555–1563. doi:10.1128/AAC.00854-09. 20124000 PMC2849361

[39] Verwer PE, van Duijn ML, Tavakol M, Bakker-Woudenberg IA, van de Sande WW 2012 Reshuffling of Aspergillus fumigatus cell wall components chitin and beta-glucan under the influence of caspofungin or nikkomycin Z alone or in combination. Antimicrob Agents Chemother 56:1595–1598. doi:10.1128/AAC.05323-11. 22203603 PMC3294952

[40] Pavie J, Lacroix C, Hermoso DG, Robin M, Ferry C, Bergeron A, Feuilhade M, Dromer F, Gluckman E, Molina JM, Ribaud P 2005 Breakthrough disseminated Aspergillus ustus infection in allogeneic hematopoietic stem cell transplant recipients receiving voriconazole or caspofungin prophylaxis. J Clin Microbiol 43:4902–4904. doi:10.1128/JCM.43.9.4902-4904.2005. 16145172 PMC1234079

[41] Madureira A, Bergeron A, Lacroix C, Robin M, Rocha V, de Latour RP, Ferry C, Devergie A, Lapalu J, Gluckmana E, Socie G, Ghannoum M, Ribaud P 2007 Breakthrough invasive aspergillosis in allogeneic haematopoietic stem cell transplant recipients treated with caspofungin. Int J Antimicrob Agents 30:551–554. doi:10.1016/j.ijantimicag.2007.07.026. 18029149

[42] Wetzstein GA, Green MR, Greene JN 2007 Mould breakthrough in immunosuppressed adults receiving anidulafungin: a report of 2 cases. J Infect 55:e131–3. doi:10.1016/j.jinf.2007.08.003. 17900699

[43] Antachopoulos C, Meletiadis J, Sein T, Roilides E, Walsh TJ 2008 Comparative *in vitro* pharmacodynamics of caspofungin, micafungin, and anidulafungin against germinated and nongerminated Aspergillus conidia. Antimicrob Agents Chemother 52:321–328. doi:10.1128/AAC.00699-07. 17938191 PMC2223904

[44] Eschertzhuber S, Velik-Salchner C, Hoermann C, Hoefer D, Lass-Florl C 2008 Caspofungin-resistant Aspergillus flavus after heart transplantation and mechanical circulatory support: a case report. Transpl Infect Dis 10:190–192. doi:10.1111/j.1399-3062.2007.00252.x. 17605738

[45] Lafaurie M, Lapalu J, Raffoux E, Breton B, Lacroix C, Socie G, Porcher R, Ribaud P, Touratier S, Molina JM 2010 High rate of breakthrough invasive aspergillosis among patients receiving caspofungin for persistent fever and neutropenia. Clin Microbiol Infect 16:1191–1196. doi:10.1111/j.1469-0691.2009.03050.x. 19735276

[46] Pang KA, Godet C, Fekkar A, Scholler J, Nivoix Y, Letscher-Bru V, Massias L, Kauffmann-Lacroix C, Elsendoorn A, Uzunov M, Datry A, Herbrecht R 2012 Breakthrough invasive mould infections in patients treated with caspofungin. J Infect 64:424–429. doi:10.1016/j.jinf.2011.12.015. 22227384

[47] Lamoth F, Juvvadi PR, Gehrke C, Steinbach WJ 2013 *In vitro* activity of calcineurin and heat shock protein 90 inhibitors against Aspergillus fumigatus azole- and echinocandin-resistant strains. Antimicrob Agents Chemother 57:1035–1039. doi:10.1128/AAC.01857-12. 23165466 PMC3553695

[48] Morris SK, Allen UD, Gupta S, Richardson SE 2012 Breakthrough filamentous fungal infections in pediatric hematopoetic stem cell transplant and oncology patients receiving caspofungin. Can J Infect Dis Med Microbiol 23:179–182. 24294271 10.1155/2012/957973 PMC3597394

[49] Gardiner RE, Souteropoulos P, Park S, Perlin DS 2005 Characterization of Aspergillus fumigatus mutants with reduced susceptibility to caspofungin. Med Mycol 43(Suppl):S299–S305. 16110824 10.1080/13693780400029023

[50] Rocha EM, Garcia-Effron G, Park S, Perlin DS 2007 A Ser678Pro substitution in Fks1p confers resistance to echinocandin drugs in Aspergillus fumigatus. Antimicrob Agents Chemother 51:4174–4176. doi:10.1128/AAC.00917-07. 17724146 PMC2151465

[51] Arendrup MC, Garcia-Effron G, Buzina W, Mortensen KL, Reiter N, Lundin C, Jensen HE, Lass-Florl C, Perlin DS, Bruun B 2009 Breakthrough Aspergillus fumigatus and Candida albicans double infection during caspofungin treatment: laboratory characteristics and implication for susceptibility testing. Antimicrob Agents Chemother 53:1185–1193. doi:10.1128/AAC.01292-08. 19104024 PMC2650576

[52] Stevens DA, Ichinomiya M, Koshi Y, Horiuchi H 2006 Escape of Candida from caspofungin inhibition at concentrations above the MIC (paradoxical effect) accomplished by increased cell wall chitin; evidence for beta-1,6-glucan synthesis inhibition by caspofungin. Antimicrob Agents Chemother 50:3160–3161. doi:10.1128/AAC.00563-06. 16940118 PMC1563524

[53] Rogg LE, Fortwendel JR, Juvvadi PR, Lilley A, Steinbach WJ 2011 The chitin synthase genes *chsA* and *chsC* are not required for cell wall stress responses in the human pathogen Aspergillus fumigatus. Biochem Biophys Res Commun 411:549–554. doi:10.1016/j.bbrc.2011.06.180. 21763289 PMC3712863

[54] Kim KW, Hyun JW 2007 Nonhost-associated proliferation of intrahyphal hyphae of citrus scab fungus Elsinoe fawcettii: refining the perception of cell-within-a-cell organization. Micron 38:565–571. doi:10.1016/j.micron.2006.10.007. 17137785

[55] Kelly J, Rowan R, McCann M, Kavanagh K 2009 Exposure to caspofungin activates Cap and Hog pathways in Candida albicans. Med Mycol 47:697–706. doi:10.3109/13693780802552606. 19888802

[56] Wiederhold NP, Kontoyiannis DP, Prince RA, Lewis RE 2005 Attenuation of the activity of caspofungin at high concentrations against Candida albicans: possible role of cell wall integrity and calcineurin pathways. Antimicrob Agents Chemother 49:5146–5148. doi:10.1128/AAC.49.12.5146-5148.2005. 16304189 PMC1315970

[57] Sandovsky-Losica H, Shwartzman R, Lahat Y, Segal E 2008 Antifungal activity against Candida albicans of nikkomycin Z in combination with caspofungin, voriconazole or amphotericin B. J Antimicrob Chemother 62:635–637. doi:10.1093/jac/dkn216. 18490373

[58] Steinbach WJ, Cramer RA Jr, Perfect BZ, Henn C, Nielsen K, Heitman J, Perfect JR 2007 Calcineurin inhibition or mutation enhances cell wall inhibitors against Aspergillus fumigatus. Antimicrob Agents Chemother 51:2979–2981. doi:10.1128/AAC.01394-06. 17502415 PMC1932494

[59] Ganesan LT, Manavathu EK, Cutright JL, Alangaden GJ, Chandrasekar PH 2004 *In-vitro* activity of nikkomycin Z alone and in combination with polyenes, triazoles or echinocandins against Aspergillus fumigatus. Clin Microbiol Infect 10:961–966. doi:10.1111/j.1469-0691.2004.00996.x. 15521997

[60] Stevens DA 2000 Drug interaction studies of a glucan synthase inhibitor (LY 303366) and a chitin synthase inhibitor (nikkomycin Z) for inhibition and killing of fungal pathogens. Antimicrob Agents Chemother 44:2547–2548. doi:10.1128/AAC.44.9.2547-2548.2000. 10952614 PMC90104

[61] Fernandes C, Anjos J, Walker LA, Silva BM, Cortes L, Mota M, Munro CA, Gow NA, Goncalves T 2014 Modulation of Alternaria infectoria cell wall chitin and glucan synthesis by cell wall synthase inhibitors. Antimicrob Agents Chemother 58:2894–2904. doi:10.1128/AAC.02647-13. 24614372 PMC3993226

[62] Chiou CC, Mavrogiorgos N, Tillem E, Hector R, Walsh TJ 2001 Synergy, pharmacodynamics, and time-sequenced ultrastructural changes of the interaction between nikkomycin Z and the echinocandin FK463 against Aspergillus fumigatus. Antimicrob Agents Chemother 45:3310–3321. doi:10.1128/AAC.45.12.3310-3321.2001. 11709302 PMC90831

[63] Clemons KV, Stevens DA 2006 Animal models testing monotherapy versus combination antifungal therapy: lessons learned and future directions. Curr Opin Infect Dis 19:360–364. doi:10.1097/01.qco.0000235163.70678.59. 16804384

[64] Luque JC, Clemons KV, Stevens DA 2003 Efficacy of micafungin alone or in combination against systemic murine aspergillosis. Antimicrob Agents Chemother 47:1452–1455. doi:10.1128/AAC.47.4.1452-1455.2003. 12654692 PMC152509

