# Supplementary material for: Caspofungin Treatment of Aspergillus fumigatus Results in ChsG-Dependent Upregulation of Chitin Synthesis and the Formation of Chitin-Rich Microcolonies
Source: Antimicrob Agents Chemother. 2015 Sep 18;59(10):5932–41. doi: 10.1128/AAC.00862-15 (PMC4576100; doi:10.1128/AAC.00862-15)
Supplement: Supplemental material [file supp_59_10_5932__index.html]

Caspofungin Treatment of Aspergillus fumigatus Results in ChsG-Dependent Upregulation of Chitin Synthesis and the Formation of Chitin-Rich Microcolonies — Supplemental material 

# Caspofungin Treatment of Aspergillus fumigatus Results in ChsG-Dependent Upregulation of Chitin Synthesis and the Formation of Chitin-Rich Microcolonies

## Supplemental material

- Supplemental file 1 -

  Supplemental Movie S1: formation of intrahyphal hyphae and newly formed septum in germlings of *A. fumigatus* treated with 32 μg/ml caspofungin.

  AVI, 2.9M
- Supplemental file 2 -

  Supplemental Movie S2: formation of intrahyphal hyphae and newly formed septum in germlings of *A. fumigatus* pregrown in medium containing 200 mM CaCl2 and 100 μg/ml CFW prior to caspofungin treatment.

  AVI, 880K
- Supplemental file 3 -

  Supplemental Movie S3: *A. fumigatus* forms wide hyperbranched hyphae in response to caspofungin treatment.

  AVI, 2.3M
- Supplemental file 4 -

  Supplemental Movie S4: treatment of *A. fumigatus* germlings with 32 μg/ml caspofungin resulting in a compensatory increase in chitin content in hyphal regions, distal-to-tip lysis, and the growth of wide hyperbranched hyphae.

  AVI, 560K
